# Hereditary Optic Neuropathies: Induced Pluripotent Stem Cell-Based 2D/3D Approaches

**DOI:** 10.3390/genes12010112

**Published:** 2021-01-18

**Authors:** Marta García-López, Joaquín Arenas, M. Esther Gallardo

**Affiliations:** 1 Grupo de Investigación Traslacional con Células iPS, Instituto de Investigación Sanitaria Hospital 12 de Octubre (i+12), 28041 Madrid, Spain; martagl.imas12@h12o.es; 2 Grupo de Enfermedades Raras, Mitocondriales y Neuromusculares, Instituto de Investigación Sanitaria Hospital 12 de Octubre (i+12), 28041 Madrid, Spain; joaquin.arenas@salud.madrid.org; 3 Centro de Investigación Biomédica en Red (CIBERER), Madrid, Spain

**Keywords:** induced pluripotent stem cells, iPSCs, iPS, optic neuropathies, optic atrophy, mitochondriopathy, retinal ganglion cells, organoids, tissue engineering

## Abstract

Inherited optic neuropathies share visual impairment due to the degeneration of retinal ganglion cells (RGCs) as the hallmark of the disease. This group of genetic disorders are caused by mutations in nuclear genes or in the mitochondrial DNA (mtDNA). An impaired mitochondrial function is the underlying mechanism of these diseases. Currently, optic neuropathies lack an effective treatment, and the implementation of induced pluripotent stem cell (iPSC) technology would entail a huge step forward. The generation of iPSC-derived RGCs would allow faithfully modeling these disorders, and these RGCs would represent an appealing platform for drug screening as well, paving the way for a proper therapy. Here, we review the ongoing two-dimensional (2D) and three-dimensional (3D) approaches based on iPSCs and their applications, taking into account the more innovative technologies, which include tissue engineering or microfluidics.

## 1. Introduction

Hereditary optic neuropathies are a heterogeneous group of disorders mainly characterized by progressive visual impairment and optic atrophy, with an estimated prevalence of 1 in 10,000 individuals [1]. These disorders share mitochondrial dysfunction as their primary pathophysiological mechanism, which causes the degeneration of retinal ganglion cells (RGCs) and their axons, leading to optic nerve atrophy [2]. The degeneration manifests in typically bilateral, symmetrical, reduced visual acuity and color vision defects, with blindness in many cases [3]. Current treatments for inherited optic neuropathies consist of pharmacological approaches, including neurotrophic factors to prevent RGC apoptosis and compounds to diminish oxidative damage, such as idebenone [4,5]. Idebenone has been approved by the European Medicine Agency for the treatment for Leber’s hereditary optic neuropathy (LHON) and, currently, is the only available drug therapy for this disease, regardless of the causing mutation. It has been shown that the effect of the treatment with idebenone is persistent in patients who respond, even after therapy is terminated [6]. Remarkably, Yu-Wai-Man et al. provided both clinical and preliminary experimental evidence for a bilateral effect of unilateral intravitreal injections of a recombinant replication-defective adeno-associated virus targeting RGCs of LHON patients [7]. These findings could have major implications for gene therapy clinical trial design and outcome measures. However, the effectiveness of most of these therapies turns out to be inefficient when a critical number of cells is damaged [8]; thus, additional strategies need to be explored in order to replace RGCs that have been lost.

Since the discovery of induced pluripotent stem cells (iPSCs), many expectations have emerged, and iPSCs have created a range of possibilities for new cell-based therapies in regenerative medicine [9]. These cells were generated, for the first time, by reprogramming murine fibroblasts into a pluripotent state with the retroviral delivery of four factors: Oct3/4, Klf4, c-Myc and Sox2 [10]. One year later, the same group was able to generate iPSCs from human fibroblasts using the same transcription factors [11]. At the same time, another group was also able to achieve human iPSCs [12]. iPSCs exhibit the same morphological features and growth properties as embryonic stem cells (ESCs), but they are associated with less ethical concerns [13]. Their ability to proliferate indefinitely, along with the possibility of virtually giving rise to any cell type of the three germ layers, provide an unlimited cell source with plasticity for numerous applications [14,15].

The discovery of iPSCs technology has made a breakthrough in the biomedical field. Growing expectancy is accumulating for their usage both in drug discovery and in clinical applications. In the specific case of optic neuropathies, this technology would enable the generation of patient-specific iPSCs, followed by their directed differentiation to the affected target cell type (RGCs). RGCs would constitute a very suitable model for understanding the underlying physiopathological mechanisms of the disease and for the evaluation of novel therapeutics [16]. Moreover, iPSCs could be used in regenerative medicine through a variety of different approaches, including autologous therapies, the CRISPR/Cas9 system to edit genomic DNA at a precise locus in iPSCs or high-throughput drug screening assays [17,18]. Recently, 3D techniques have appeared, providing more accurate 3D cellular in vitro systems with the intent of faithfully recapitulating retinal development; these techniques include organoid technology and tissue engineering [19].

In this review, we recapitulate the foremost applications of iPSC-derived RGCs in the context of hereditary optic neuropathies, comprising two-dimensional (2D) and three-dimensional (3D) approaches, and the concrete examples already conducted to date.

## 2. Vulnerability of RGCs in Inherited Optic Neuropathies

*Main Pathological Features of Inherited Optic Neuropathies*

Although inherited optic neuropathies represent a heterogeneous group affecting RGCs, all these disorders share several clinical manifestations. These include usually symmetrical, bilateral and central visual loss, dyschromatopsia and a selective loss of retinal nerve fibers in the papillomacular bundle, a standard feature of optic atrophy [20]. Moreover, mitochondrial dysfunction turns out to be the underlying pathophysiological mechanism in all of them.

Leber’s hereditary optic neuropathy (LHON) and autosomal dominant optic atrophy (ADOA) are the most common non-syndromic forms within this group of disorders in society, with an approximate prevalence ranging 1/25,000 to 1/50,000 [21,22]. LHON predominantly affects young males in the second or third decade of life, provoking abnormalities such as microangiopathy and vascular tortuosity of the retinal vessels [23]. Affected individuals with LHON often lose central vision in one eye, followed by similar involvement in the other eye, and the visual acuity is deteriorated below 20/200 levels within few months [24]. Regarding genetics, in 90% of cases, LHON is caused by three homoplasmic point mutations in the mitochondrial DNA (mtDNA): m.11778G>A in the *MT*-*ND4* gene (the most common one), m.3460G>A in *MT*-*ND1* and m.14484T>C in *MT*-*ND6* [25]. All these mutations disrupt different complex I subunits of the mitochondrial respiratory chain, which prompts impaired oxidative phosphorylation and decreased ATP production. Energy failure, in combination with the accumulation of reactive oxygen species (ROS), bring about RGC vulnerability to undergo apoptosis [26]. However, the mtDNA mutations are necessary but occasionally insufficient to trigger the disease, since some individuals harboring these mutations appear not to develop LHON. The hypothesis stating the existence of additional factors that influence LHON penetrance has been largely studied, since environmental factors, a mitochondrial haplogroup or nuclear DNA background appear to have a role in LHON onset [27]. The X chromosome has been the main candidate in the search for a modifier gene, because it would also explain the male prevalence of the disease [28], but the studies have been inconclusive, not finding a causative gene. To date, exploration of different loci has led to the identification of different nuclear modifier genes [29] and even, in some cases, to the report of the concrete genetic variants [30,31]. Nevertheless, the full complex mechanism underlying LHON has not been elucidated yet.

Most LHON patients suffer from optic neuropathy as the isolated clinical manifestation of the disease. However, there are also patients that have extraocular symptoms such as movement disorders, tremors and cardiac conduction defects, among others. In these individuals, the condition is described as LHON “plus”, and genetically, it is also considered a mitochondrial disease. [32]. Some affected LHON “plus” individuals develop features similar to multiple sclerosis, frequently triggered by point mutations in complex I subunits [33].

On the other side, ADOA—also known as Kjer’s optic atrophy—was first described by the ophthalmologist Paul Kjer at the end of the 19th century [34]. ADOA is typically diagnosed in the first two decades of life, marked by a milder progressive course and characterized by a bilateral pallor of the optic disc. The clinical expression is highly variable among patients. Nonetheless, visual acuity most often remains better than 20/200 [35]. In about 60–80% of the cases, patients with ADOA present mutations in *OPA1*, a nuclear gene located on chromosome 3q28-q29. *OPA1* encodes for a ubiquitously expressed GTPase-related dynamin, which is anchored to the mitochondrial inner membrane [36,37]. This multifunctional protein plays a key role in the structural organization of the mitochondria network regulating mitochondrial fusion. Besides, it is also involved in cell survival, oxidative phosphorylation and the maintenance of mitochondrial DNA (mtDNA) [38]. To date, more than 400 *OPA1* pathogenic variants have been reported of which, 28% are missense variations, 24% are associated with altered splicing, 22% are frameshift variants, 15% are nonsense variants and 7% are deletions. Conversely, the majority of the variants result in a truncated protein, supporting haploinsufficiency as the main pathological mechanism of the disease [39]. Due to this fact, mitochondria become disarranged, and, finally, the energy production capacity is diminished. It is noteworthy that ADOA, likewise, presents incomplete penetrance, but this fact still remains poorly understood [27]. Less commonly, mutations in other loci are associated with pure DOA, the main ones being OPA4 and OPA5. Additional loci have been identified as responsible for optic atrophy, but either with an X-linked mode of inheritance like OPA2 or a recessive transmission like OPA6 and OPA7 [40]. In the last case, causative mutations have been found in *TMEM126A*. This gene encodes for a transmembrane mitochondrial protein with an unknown function [41].

DOA patients can also exhibit optic atrophy followed by sensorineural deafness, ataxia, myopathy and chronic ophthalmoplegia. This condition, described as DOA “plus” syndrome is also due to pathogenic mutations in *OPA1*, standing out in patients harboring the specific mutation c.1334G>A; p.Arg445His [42]. Compound heterozygous mutations in the same gene have been associated with Behr syndrome, characterized by optic atrophy and spinocerebellar degeneration [43]. Moreover, heterozygous mutations in *OPA3* rarely provoke an optic atrophy associated with premature cataracts, more frequently causing Costeff syndrome, an autosomal recessive disorder characterized by optic atrophy and 3-methylglutaconic aciduria type III [44]. Optic atrophy, associated with deafness, can also occur due to mutations in *OPA8* [45]. Another example is Wolfram syndrome, marked by optic atrophy, diabetes and deafness, in which autosomal recessive mutations in *WFS1* and *CISD2* are found [46]. Heterozygous mutations in the mitochondrial aconitase 2 (ACO2), an enzyme of the tricarboxylic acid cycle, have also been found to cause either isolated or syndromic optic atrophy [47]. Additionally, in some mitochondrial neurodegenerative disorders, clinical manifestations can include optic neuropathy. Among them, it is worth emphasizing Charcot-Marie Tooth type 2A, Friedreich ataxia, hereditary spastic paraplegia 7 and mitochondrial encephalomyopathies (such as MELAS or Leigh syndrome) [48].

Besides LHON and DOA, glaucoma has elicited a steady controversy about belonging to this group of hereditary optic neuropathies or not. Glaucoma is the leading cause of irreversible blindness worldwide, comprising a group of disorders in which the optic nerve degenerates, resulting in pathological disc cupping [49]. An abnormally elevated intraocular pressure is traditionally considered a hallmark in most forms of the pathology, which is closely related with RGC death [50]. Only specific types of glaucoma clearly have a genetic basis, the estimation of the genetic contribution being as high as 50–60%. Glaucoma can occur at all ages. Early-onset glaucoma (before age 40) more likely exhibits Mendelian inheritance associated with single genes, whereas the adult-onset form is inherited as a complex trait [51]. In the juvenile subtype, mostly genetic variants in *MYOC*, *CYP1B1*, *OPTN* and *TBK1* have been described, but simple mutations represent a small proportion of total glaucomatous optic neuropathy cases [52,53]. Strong evidence suggests glaucoma to be presumably a complex genetically heterogeneous disorder. In a greater percentage of cases, it may be modulated by the interaction of polymorphisms in multiple genes and environmental factors [54].

*Mitochondrial Dysfunction as a Key Player for RGC Degeneration*

**Figure 1:**
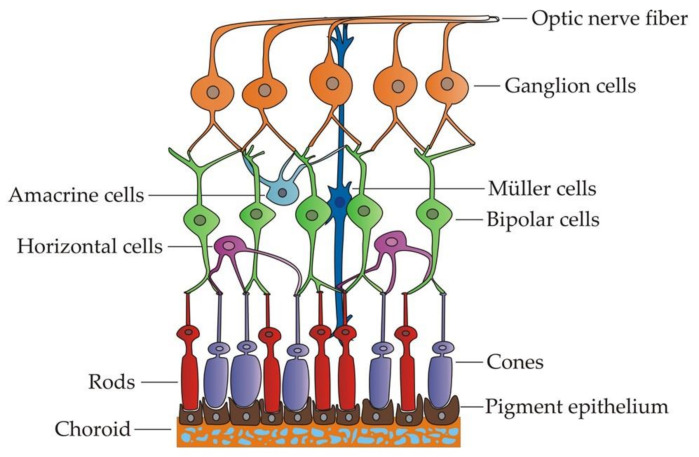
Distribution of the different cell types within the retina.

RGCs are specialized projection neurons situated near the inner surface of the retina that receive visual information from photoreceptors through bipolar and amacrine cells (Figure 1). They transform the information into action potentials and transmit them through their axons, forming the optic nerve towards the brain [55]. Hence, these cells need a high and constant energy supply to carry out their functions correctly. To this aim, RGCs rely on the mitochondria, the powerhouses of the cell, responsible for ATP production through oxidative phosphorylation [56]. For that reason, RGCs are particularly vulnerable to mitochondrial dysfunction. In addition, their unique axonal structure may also contribute to their increased susceptibility to mitochondrial damage [57]. The RGC axons only acquire a myelin sheath beyond the lamina cribosa while a long intraretinal portion remains unmyelinated. This means that the generation of action potentials in this section is less efficient and necessitates further ATP consumption [58]. Thus, the prelaminar section contains a higher concentration of mitochondria, and a decreased density would significantly limit the energy input required, particularly in conditions of chronic stress [59].

As previously described, hereditary optic neuropathies share a mitochondrial bioenergetic failure. This is caused by either mtDNA mutations that disrupt some electron transport chain (ETC) complex or mutations in nuclear genes coding essential proteins involved in the maintenance of the mitochondrial network [60]. A direct consequence of bioenergetics impairment is the incomplete oxidation of the molecular oxygen, which leads to the generation of ROS. The resulting oxidative stress is known to impair normal electron flow in the ETC, causing damage to the mitochondrial membranes, proteins and mtDNA, therefore compromising RGC viability [61]. The maintenance of RGCs is heavily dependent upon normal mitochondrial function, since, apart from energy production, mitochondria regulate diverse physiological processes that are essential for neuronal functioning [62]. In order to meet all these cellular demands, axonal transport is necessary to preserve the correct gradient of mitochondria, ensuring their presence at sites of high energy consumption. The complex pattern of mitochondrial motility results from interactions between microtubules and motor proteins, which allow their transport from the cell body toward the apical end (anterograde) and in the other direction (retrograde) [63]. Axonal transport requires ATP itself; thus, in the context of energy production impairment, it may be inhibited and the mitochondrial gradient disrupted. Additionally, a reduced motility is expected to affect the mitochondrial dynamics of fusion and fission. Mitochondria are part of a dynamic network that has to be in constant flux to achieve a balance between the processes of fusion, fission, biogenesis and mitophagy, in this way controlling cellular homeostasis [64]. The disturbance of this equilibrium, along with an altered respiratory function and elevated oxidative stress, finally prompts the apoptosis of RGCs.

## 3. Applications of iPSC-Derived RGCs

As aforementioned, iPSC-derived RGCs represent an extremely valuable tool both in the understanding of the mechanisms responsible for hereditary optic neuropathies and in the designing of clinical approaches. In order to differentiate iPSCs towards RGCs, the phases of embryonic development must be known as a means to mimicking in vivo retinal cell fate specification. Hence, a combination of different growth factors and pharmacologic agents can be employed to control signaling events and to direct iPSCs towards the retinal precursor cell lineage and, ultimately, RGC generation [65].

*RGC Specification within the Context of Retinal Development*

During the early stages of development, neural induction initiates neuroectoderm formation. Later on, the initial neural epithelium—called the neural plate—is regionalized to give rise to different neural domains. Between them, the forebrain, which originated during gastrulation from the anterior neuroectoderm, is further specified into regional subdomains, including the presumptive eye [66,67]. This region contains the earliest eye precursors and is characterized by the expression of several eye field transcription factors (EFTFs), including Rx, Six6, Otx2, Six3, Pax6, Lhx2 and Chx10 [68]. All these processes related with eye field patterning are accurately regulated by several extracellular signaling molecules, including fibroblast growth factors (FGFs), insulin-like growth factors (IGFs), retinoic acid (RA) and Sonic hedgehog (Shh). Moreover, the concomitant inhibition of the bone morphogenetic protein (BMP), Nodal and Wnt/ß-catenin signaling pathways in the anterior neural plate are required [69].

**Figure 2:**
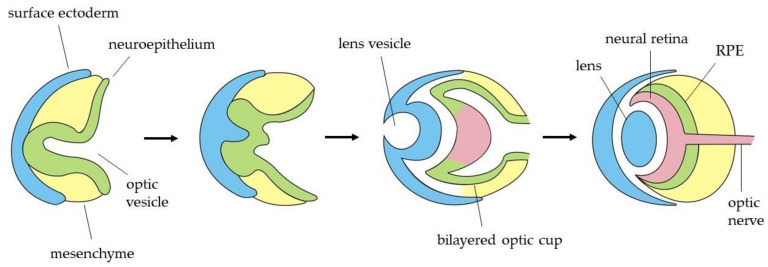
Temporal sequence for the formation of the optic cups, containing both the retinal pigment epithelium (RPE) and the neural retina.

Shortly after the eye field specification, these EFTFs induce the region to evaginate bilaterally to form two optic vesicles (OVs) (Figure 2). The distal part of the OV, which is the presumptive retina, interacts with the overlying surface ectoderm, leading to an invagination to form both the lens vesicle and the bilayered optic cup [70]. Subsequently, the outer layer establishes the retinal pigmented epithelium (RPE), while the inner layer proliferates extensively to give rise to the neural retina (NR), which contains multipotent retinal progenitor cells (RPCs). Optic cup layers organization is guided by two regulatory molecules: Mitf as a crucial transcription factor for the acquisition and maintenance of RPE cell identity and Chx10 as the main regulator of neuroretinal development. Both genes function in an antagonistic way in the specification of these cells, each one inhibiting the other one expression in the corresponding region [71]. Thereafter, RPCs in the neural retina differentiate, in a temporally conserved sequence, to develop the different cell types of the adult retina, which are the retinal ganglion cells, cone photoreceptors, horizontal cells, amacrine cells, rod photoreceptors, bipolar cells and Müller glia [72]. All these cells become organized into three nuclear layers in the adult retina. The outer nuclear layer contains the photoreceptor cell bodies, which project their segments toward the adjacent RPE. Photoreceptors receive visual information, and they connect with bipolar, horizontal and amacrine cells situated in the inner nuclear layer. Finally, the information gets to the RGCs, placed in the innermost layer (also known as retinal ganglion cell layer), which are the cells responsible to transmit it to specific targets in the brain towards the optic nerve [73].

RGCs are the first neuronal cell type to arise in the retina. Their differentiation is regulated by several transcription factors. Firstly, Atoh7 regulates RGC fate determination, and its expression is coincident with RGC emerging [74]. This intrinsic factor regulates a network of RGC-specific genes, such as *Brn3b* and *Islet-1*, which are necessary for the development of specific subpopulations of RGCs [75]. In addition to them, the Notch pathway acts as a negative regulator, which is downregulated prior to RGC specification [76]. Knowledge of all the pathways would allow the designing of specific protocols for iPSC differentiation towards an RGC fate.

### 3.1. 2D Approaches

Conventional 2D culture procedures to differentiate iPSCs towards RGCs usually involve the formation of embryoid bodies (EBs), which are a mixture of cells of each primary germ layer in development [77]. Later on, the presence of an appropriate culture matrix and the addition of concrete molecules or factors would allow the EBs to form neural rosettes containing clusters of neuroprogenitor cells [78,79]. Apart from inhibitors or activators of specific pathways, it is common to use certain supplements for neural retina specification, such as the N2 and B27 pro-neural supplements. This step is usually followed by the selection of the neural rosette structure to generate neurospheres in suspension, which are proliferative aggregates of neural precursor cells with the potential to give rise to the desired population of RGCs [80].

Originally, in 2006, Lamba and colleagues set up an efficient protocol for the generation of retinal progenitor cells from human ESCs for the first time. Firstly, they generated EBs and used a combination of molecules for neural induction: Noggin (inhibitor of the BMP pathway), Dkk1 (inhibitor of the Wnt/ß-catenin pathway) and IGF-I. In about 80 days, the retinal neurons could differentiate into RGCs and amacrine cells [81]. A few years later, Tucker et al. performed a similar protocol to differentiate iPSCs towards the retinal lineage using a xeno-free synthetic culture matrix. Between other cell types, they found some cells that were positive for RGC markers [82].

Later on, the differentiation protocols focused on specific RGC generation rather than a mixture of retinal cell types. The main features of these iPSC-derived RGC protocols are summarized in Table 1. In 2014, Riazifar et al. published a quite simple protocol in which neural rosettes were generated plating EBs in a gelatin substrate, and, subsequently, rosettes were selected for neurosphere formation. The addition of the Notch inhibitor N-(N-(3,5-difluorophenacetyl)-L-alanyl)-S-phenylglycine t-butyl ester (DAPT) allowed RGC generation in 40 days with an efficiency of around 20–30%. This was assessed by the expression of the markers Islet-1, TUJ-1, γ-synuclein, BRN3A and THY1 [83]. The following year, Tanaka et al. [84] generated self-induced RGCs by adapting a two-stage protocol previously published by Nakano et al. in 2012 for the formation of neural retina using ESCs [85]. The first part of the approach consisted of a suspension culture for EB generation in a retinal differentiation medium, with the sequential addition of the Wnt inhibitor (IWR-1e), fetal bovine serum (FBS), SAG (Shh agonist) and CHIR99021 (Wnt agonist). It was followed by a period of adherent culture when the aggregates were transferred to poly-D-lysine/laminin-coated plates. In this stage, the medium was changed to a retinal maturation medium containing a N2 supplement and the addition of retinoic acid. The protocol took 35 days, but the differentiation efficiency was not reported [84]. Shortly after, Ohlemacher et al. accomplished iPSCs-derived RGC identification using a stepwise differentiation protocol. The procedure started culturing EBs in a neural induction medium containing a N2 supplement. These EBs were induced to adhere with the addition of fetal bovine serum (FBS). Then, neurospheres were generated in suspension culture, maintained in a retinal differentiation medium with a B27 supplement and, finally, enriched for further differentiation. In 40 days, Brn3-positive RGCs comprised approximately 36%, but, around 70 days, were required for RGC maturation [86]. In 2017, Teotia et al. reported RGC generation starting from iPSC-derived RPCs on the basis of the retinal induction protocol published by Lamba and colleagues [81]. The generated RPCs were plated on Matrigel and cultured with the stage-specific inducers Shh, FGF8, follistatin, cyclopamine and DAPT. In 15 days, around 20–30% of RPC-derived RGCs co-expressed Brn3 and Tuj1 [87]. One year later, Lee et al. established another method for RGC generation from iPSCs. The first step of the procedure was the induction of eye field progenitors by inhibiting the required pathways with dorsomorphin (BMP inhibitor), SB431542 (TGF-β inhibitor) and XAV939 (Wnt inhibitor), along with the addition of IGF-I. Then, neural rosettes were formed in the Matrigel substrate and, subsequently, selected in order to enrich retinal progenitors. After a Notch inhibition with DAPT, RGC progenitors were obtained and further differentiated and maturated towards the final RGC population. In about 40 days, they got 45% of the BRN3B and ISLET1 double-positive RGCs [88]. Recently, Chavali et al. presented another RGC differentiation methodology adapted from several previous protocols. It included the inhibition of the three signaling pathways described by Lee et al., followed by the culture with the chemically defined media standardized by Teotia et al. with several modifications. They generated over 80% of the pure RGC cultures in about 40 days and purified the Thy1+ cells thanks to the unique expression of the surface glycoprotein Thy1 in the RGCs. Employing magnetic-activated cell sorting (MACS) and CD90.2 microbeads, they obtained 95% of the cells positive for BRN3A [89]. In all these differentiation protocols, elongated axons were observed. The RGC functionality was also demonstrated, whether through the assessment of axonal transport or the generation of action potentials.

Other published 2D differentiation protocols into RGCs are adapted methodologies from these original ones or even combinations among several of them. A hurdle to overcome is that, typically, the differentiation procedure yields a heterogeneous population of differentiated cells containing RGCs but, also, other cell types. When the ratio is not high enough, procedures need to incorporate a purification or enrichment step to isolate the RGC population. For instance, similarly to Chavali et al. [89], Gill and colleagues employed MACS to isolate THY1 positive human ESC (hESC)-derived RGCs [90]. Alternatively, Sluch et al. purified RGCs differentiated from hESCs using a CRISPR-engineered RGC fluorescence reporter stem cell line by means of fluorescence-activated cell sorting [91]. Additionally, the modification in the differentiation protocol developed by Hsu’s group in 2019 remarkably obtained a more homogeneous population of RGCs. Once neurospheres were obtained, they harvested and dissociated them into single cells and used differential adhesion properties of the different cell types contained in the neurospheres. Generally, RGCs are the first cell type that appear in differentiating neurospheres and they present a lower adherence than other cell types. Single cells were seeded briefly onto Geltrex-coated plates, and unattached RGCs were collected from the supernatant for further differentiation [92].

*Potential Applications for iPSC-Derived RGCs*

The establishment of a robust and reproducible differentiation protocol could represent a highly valuable tool for modeling hereditary optic neuropathies. iPSC-derived RGCs could recapitulate the main features of these diseases in vitro, helping us to understand the underlying physiological mechanisms of RGC loss [93]. Moreover, they would provide a unique platform for the study of emerging therapeutic approaches, such as drug screening strategies to identify compounds able to rescue the cellular defect [94]. In this regard, different research groups have already generated iPSC lines from the somatic cells of patients with optic neuropathies, harboring mutations in genes that have been described as being associated with them. Although the use of iPSC-derived RGCs in high-throughput screening studies has not been reported yet, some groups have been able to find molecules that potentially rescue the affected phenotype. For instance, iPSC lines were established from fibroblasts of normal-tension glaucoma patients with *TBK1* gene duplication. This allowed the demonstration that iPSC-derived RGCs had increased levels of LC3-II (a lipidated form of microtubule-associated protein 1A/1B-light chain 3), a key marker of autophagy, and thus, this pathway was identified to be dysregulated [95]. Besides, fibroblasts from a patient with glaucoma possessing a p.Glu50Lys missense mutation in the OPTN gene were also reprogrammed for iPSCs, followed by their differentiation towards RGCs [86]. Enhanced caspase-3 activation in the affected RGCs was observed in comparison with control iPSC-derived RGCs, indicative of increased levels of apoptosis. They identified brain-derived neurotrophic factor (BDNF) and pigment epithelial-derived factor (PEDF) as neuroprotective factors with the capacity to significantly reduce caspase-3 activation [86].

Other iPSC lines were generated from fibroblasts obtained from patients with an optic atrophy “plus” phenotype: one carrying the mutation c.1861C>T; p.Gln621Ter in *OPA1* [96] and the other one harboring the mutation c.1635C>A; p.Ser545Arg in *OPA1*, both of them in heterozygosis [97]. Likewise, fibroblasts from patients diagnosed with optic atrophy, carrying an intronic mutation in *OPA1* (intron 24 c.2496+1G>T), were reprogrammed into the iPSCs. The authors found that the mutation caused an increase in apoptosis in iPSCs, and they were unable to differentiate into RGCs. Noggin and β-estrogen were identified as potential therapeutic agents, since these compounds were able to rescue the phenotype [98]. Fibroblasts harboring mutations in other genes causing dominant optic atrophy have also been reprogrammed. For instance, an iPSC line was obtained from a patient’s fibroblasts carrying the heterozygous mutation c.1999G>A; p.Glu667Lys in *ACO2* [99].

LHON patient iPSCs have been generated as well. In one case, fibroblasts from a patient carrying the homoplasmic primary mutation in the *MT*-*ND1* gene (m.3460G>A; p.Ala52Thr) were reprogrammed [100]. Other iPSC lines were generated from fibroblasts carrying homoplasmic double-mtDNA mutations in the *MT*-*ND1* gene (m.4160T>C; p.Leu285Pro) and in the *MT*-*ND6* gene (m.14484T>C; p.Met464Val) (in this case, triggering the LHON “plus” phenotype), along with fibroblasts harboring the most common homoplasmic mtDNA mutation in the *MT*-*ND4* gene (m.11778G>C; p.Arg340His) [101]. The same group used transmitochondrial cybrid technology to generate an isogenic control replacing the mutant mtDNA, which was subsequently differentiated to RGCs. They observed a return of apoptosis to normal levels in the cybrid-corrected RGCs in comparison with the increased levels found in LHON iPSC-derived RGCs [102]. More groups generated iPSC models of LHON with the representative mutation m.11778G>C; p.Arg340His [103,104]. One of them, Wu et al., showed defective neurite outgrowth in RGCs differentiated from patient iPSCs and improved mitochondrial biogenesis as a compensatory mechanism. Additionally, they demonstrated in another study the existence of an impaired mitochondrial respiration in affected RGCs, which led to deficient neuronal function. The LHON-RGCs presented electrophysiological activity dysfunction, implying that the mutation could cause defects in the ionic channels [105].

Within the field of modeling hereditary optic neuropathies, genome editing technology could represent a highly advantageous tool with the likelihood of correcting the causative mutation in a given patient-specific iPSC line. In this way, isogenic-paired iPSCs could be produced using nucleases like ZFNs, TALENs or the most innovative, the CRISPR/Cas9 editing tool, which have rapidly become the prevalent system for the genetic manipulation of iPSCs [106]. Furthermore, genomic correction holds great promise in the regenerative medicine field, opening the way to personalized medicine, since RGCs could be differentiated from corrected iPSCs for their usage in autologous cell transplantation [107]. In this sense, using iPSCs for cell replacement therapy turns out to be an appealing therapeutic option for inherited optic neuropathies, particularly once significant RGC loss has occurred [108]. Up to now, some studies have provided proof-of-concept for using iPSC derivatives in order to replace degenerated RGCs using animal models for cell transplantation. In 2013, Satarian et al. prompted human iPSCs to differentiate into anterior specified neural progenitors and transplanted them intravitreally into rats whose optic nerves had been crushed. After several weeks, the grafted cells were able to integrate into the RGC layer and differentiate into the neuronal cell lineage, further showing a significant protective effect on the affected RGCs and the recovery of their function [109]. In 2015, Parameswaran et al. transplanted, for the first time, mouse iPSC-derived RGCs in a rat model of ocular hypertension. The cells integrated into the host’s RGC layer and expressed several axonal guidance molecules, suggesting their potential to functionally replace the lost RGCs [110]. However, optic nerve regeneration still remains the major challenge. The grafted cells should be able to connect with amacrine and bipolar presynaptic cells in the host retina, as well as projecting their axons through the correct targets in the brain [111]. Hence, more research into RGC in vivo transplantation is needed to assess both the correct integration and connection of differentiated neurons.

### 3.2. 3D Approaches

As discussed previously, 2D culture systems to generate iPSC-derived RGCs would entail a huge potential to model hereditary optic neuropathies and, as a last resort, to find appropriate treatments for them. However, the obtained RGCs lack the typical organization found in the retina, including their complex interactions with other retinal cells and the surrounding matrix, which could restrain their suitability for cell therapy applications. Due to these limitations, 3D cell culture technologies associated with the conventional methods have emerged, providing a similar environment to the one in the tissue [112]. Three-dimensional approaches comprise organoid technology and other recent tools, like tissue engineering and microfluidics.

*Ganglion Cell Layer Generation Using Retinal Organoid Technology*

Several research groups have differentiated iPSCs into optic cup-like retinal organoids (Table 2), allowing cells to self-organize in an ex vivo 3D structure that gives rise to most of the major retinal cell types. This way, the RGC population differentiated within the organoid recapitulates, more faithfully, the developmental stages that occur in vivo, both spatially and temporally. Moreover, once the organoid is generated, the RGCs can be positively identified and eventually isolated for clinical applications [113].

In 2011, Meyer and colleagues published, for the first time, a stepwise differentiation protocol for the generation of floating OV-like structures derived from both ESCs and iPSCs [114,115]. This procedure started with the generation of EBs in a N2-containing neural induction medium, followed by their adhesion in order to enrich the retinal progenitor cells. After that, there was a switch from adherent to free-floating culture conditions for further retinal differentiation with the addition of a B27 supplement. Finally, manual separation of the OV-like neurospheres was performed to obtain a purified population. Afterwards, this method, with some modifications, was used by Zhong et al. in 2014 for long-term culture of the OVs, including the addition of FBS and taurine. The formation of 3D retinal cups had an efficiency ranging from 50 to 70%, developing these optic cups all major retinal cell types arranged in their proper layers. RGCs (BRN3-positive) first appeared in five weeks, and the emerging ganglion cell layer became well-established in a total of 12 weeks, approximately [116]. The same year, Reichman et al. published another procedure to differentiate iPSCs into both RPE cells and neural retina-like structures using a proneural medium. The particular features of this method were the bypass of EB formation and the lower number of exogenous molecules supplied. RPCs contained in the neural retina-like structures gave rise to all retinal cell types in the floating culture, including RGCs at day 21 [117].

In 2012, Nakano et al. published another relevant pioneer method for the self-formation of optic cup structures and neural retina starting from ESCs in a serum-free medium [83]. This procedure has been extensively adapted by other research groups using iPSCs. One example of this is the xeno-free modification published by Wiley and colleagues in 2016 [118]. This modification consisted firstly of a stage of sphere formation in 3D differentiation media containing IWR1e, SAG and CHIR99021. Additionally, they included a human extracellular mixture to enhance the neural epithelium formation. In the next stage, the eye cups were formed in a neural retina culture media supplemented with N2, followed by their selection for neural retina maturation. Between weeks six and eight, HuC/D-positive retinal amacrine- and ganglion cell-like neurons were distinguished [118]. Concurrently, Lowe et al. also generated iPSC-derived retinal organoids. Epithelialized cysts were formed by floating culture clumps of iPSCs and Matrigel in a medium containing N2 and B27 supplements. Then, the cysts attached spontaneously and were detached as cell sheets that were grown as floating structures only with B27. For long-term culture, retinal organoids were cultured in the presence of taurine and FBS, and they gradually generated stratified retinal tissues, the RGCs identified by ISL1/2 and TUJ1 expression [119].

In 2018, Kobayashi and colleagues adapted a protocol from the one previously published by Kuwahara et al. [120] to generate 3D retinal organoids from iPSCs with the final aim of purifying the RGC population [121]. Briefly, they generated EBs in a medium containing a knockout serum replacement, which was supplemented with bone morphogenetic protein 4 (BMP4) to selectively prompt the formation of 3D neuroepithelium. Thereafter, retinal organoids were cultured with a N2 supplement, CHIR99021 and SU5402 (a FGF inhibitor), and finally the culture medium was replaced with a maturation medium containing N2 and FBS. RGCs were successfully identified from day 40 by BRN3B expression, and, subsequently, they purified them by a two-step immunopanning method using anti-Thy1 antibodies. These RGCs could be further cultured in a specific medium [121]. The protocols developed by other groups have been based on this original methodology but introduced some minor modifications [122,123]. The following year, Mellough et al. generated retinal organoids following a different procedure. Firstly, EBs were formed by different approaches to determine the impact of the EB formation method in retinal differentiation. They observed that the most appropriate method was mechanical EB formation under static conditions. EBs were further differentiated in the presence of IGF-I with B27 supplementation throughout and, lately, with N2 supplementation. At week five, melanopsin-photosensitive ganglion cells were detected thanks to the differential expression of *OPN4*. Moreover, developing RGCs were situated in the basal aspect and showed Smi32-immunopositive processes (the nonphosphorylated form of the neurofilament heavy chain protein, which strongly labels a RGC subtype), along with positive nuclei, for the RNA-binding protein RBPMS (also a marker for RGCs) [124]. This procedure was also applied with modifications by Chichagova and colleagues [125].

In the last years, the number of methods to produce retinal organoids from iPSCs has considerably raised, achieving the reproduction of a developmental sequence that closely simulates normal human retinogenesis [126]. Nonetheless, the reality is that some features can vary within and among organoids, meaning a relative lack of uniformity. Recently, the National Eye Institute sponsored the “3D Retinal Organoid Challenge 2020” with the aim of eliminating the limitations in the current organoid generation methods by the development of a robust 3D retinal organoid system that truly mimics the retina. In fact, Gamm’s laboratory differentiated 16 lines (ESCs and iPSCs) towards retinal organoids by applying a method based on Meyer’s protocol (2011) [114] and some adaptations of others (Zhong’s and Kuwahara’s) [116,120]. Over the course of the study, they observed three stages in all 3D cultures. Retinal organoids in stage 1 contained proliferative neural retina progenitors, which led to the formation of a robust inner RGC layer. In stage 2, the RGC layer was gradually degenerated, and the progenitors differentiated progressively into photoreceptors and horizontal and amacrine cells. Finally, stage 3 was characterized by the presence of active photoreceptors that formed specialized synapses in addition to other peculiarities of advanced retinal organization [127]. Even though these stages were easily distinguished in each organoid, and this helped to gain a better understanding of their behavior, some inconsistencies in the composition and the maturation were still present when comparing organoids generated from different lines. This is also due to the fact that several shortcomings are associated with retinal organoids, since there are evident differences between the in vitro and in vivo environments. Furthermore, generating large quantities of retinal organoids for the development of a specific transplantation strategy is a considerable long-lasting process, often leading to morphological disorganization and cell death [128]. Therefore, there is an emerging need for improving retinal organoid technology—for instance, including vascularization and nutrients perfusion or solving the problem of incomplete functional maturation [129,130].

In this respect, some research groups have recently explored the employment of bioreactors to increase the differentiation yield towards retinal organoids. As an example, DiStefano and colleagues reported a bioprocess using rotating-wall vessel bioreactors to culture retinal organoids derived from mouse ESCs [131]. In this study, the authors demonstrated that bioreactors can improve and accelerate organoid growth and differentiation for modeling retinal diseases and the evaluation of therapies. Thus, they presented this investigation as a proof-of-concept that could be easily applied for the differentiation of iPSCs [131]. The same year, Ovando-Roche et al. investigated the use of stirred-tank bioreactors for retinal organoid generation from iPSCs. The bioreactor culture conditions decreased the number of apoptotic cells in the retinal organoids while increasing the cell proliferation. Moreover, they observed an improved laminar stratification of the organoids and the growing of more complex structures [126]. Hence, although further studies are required, bioreactors could represent a promising platform for scaling up the manufacture of retinal cells, including RGCs, and permit their enrichment for an eventual translation to the clinics.

**Table 2:** Differentiation protocols of human iPSCs towards retinal organoids containing a ganglion cell layer.

Authors	Adapted Protocol	Differentiation Approach	Differentiation Factors	Culture Matrix	Markers for RGC Identification	Isolation Method	Organoids Culture Time (Weeks)	Time for RGC Differentiation (Days)	Reference
Zhong et al., 2014	Meyer et al., 2011	EBs, neural rosettes, OV-like structures	N2, B27, FBS, taurine	Matrigel (growth factor reduced)	BRN3, HUC/D, TUJ1	N/A	25	35	[116]
Reichman et al., 2014	Original	Neural retina-like structures	N2, bFGF	-	BRN3A, BRN3B	N/A	16	21	[117]
Wiley et al., 2016	Nakano et al., 2012	EBs, optic cup-like structures	IWR1e, SAG, CHIR99021, N2	-	HuC/D	N/A	11	45	[118]
Lowe et al., 2016	Original	Cysts, cell sheets, retinal organoids	Matrigel, N2, B27, taurine, FBS	-	ISL1/2, TUJ1	N/A	22	54	[119]
Kobayashi et al., 2018	Kuwahara et al., 2015	EBs, retinal organoids	BMP4, N2, CHIR99021, SU5402, FBS	-	ATOH7, BRN3B, ISL1, RBPMS, THY1	Two-step immunopanning with anti-Thy1 antibodies	17	40	[121]
Mellough et al., 2019	Original	EBs, retinal organoids	IGF-I, B27, N2	-	*OPN4*, Smi32, RBPMS	N/A	21	35	[124]

EBs, Embryoid Bodies; FBS, Fetal Bovine Serum; bFGF, Basic Fibroblast Growth Factor; SAG, Shh Agonist; BMP4, Bone Morphogenetic Protein 4; RBPMS, RNA-Binding Protein with Multiple Splicing.

Further to this, it should be noted that additional protocols for organoids generated from iPSCs focusing on RGC layer formation are needed to broadly address the issue of axon projection. Additionally, it would allow looking into the possibility of establishing connections amongst retina and the forebrain in order to enhance RGC survival [112].

*Biomaterials-Based Microfluidics to Improve iPSCs-Derived RGCs 3D Models*

Apart from organoid technology, the development of innovative tools, including tissue engineering approaches and microfluidics, has marked a major step forward in 3D culture models with iPSC-derived RGCs [19]. Different biomaterials can be used to engineer artificial scaffolds, and the progress in microfabrication techniques generates the possibility of precisely controlling their biophysical and biochemical properties. These scaffolds would provide physical support and could directionally guide the long axons of iPSC-derived RGCs, addressing the main challenge of RGC transplantation [132,133]. This way, the employment of scaffold-based delivery systems could enhance RGC survival or direct their proper differentiation, holding immense potential for the treatment of hereditary optic neuropathies [134]. In this review, we included 3D-tissue engineering approaches that use scaffolds to guide cell growth. The scaffold can be processed in different ways: some examples aim to create a pattern in the scaffold that would help to orientate the RGCs growing in a monolayer, while others use the scaffold to support cell growth in three dimensions.

Given the novelty of this field, only a few examples have been described for the engineering of RGC scaffold biomaterials from iPSCs. In 2017, Zhong’s group used their previously published procedure [116] to differentiate RGCs from the iPSC-neural retina. Then, they fabricated a biodegradable poly (lactic-co-glycolic acid) (PLGA) micro-scaffold by electrostatic spinning with a random fiber orientation to guide the RGC growth. They proved that RGCs integrated correctly with the scaffold and were capable of growing functional axons and neurites, mimicking the nerve fiber layer of the retina. The scaffold was transplanted into the retinas of rabbits and monkeys and, two weeks later, was partially attached to the retinas. Moreover, they did not observe any adverse effects three months after the surgery. However, several shortcomings were identified: the biodegradability of the scaffold should be better adjusted in future studies, and the RGCs were not fully matured; thus, a chemically defined protocol would be required [135]. The same year, Yang et al. used the basis of Ohlemacher’s differentiation protocol [86] to generate iPSC-derived OVs to give rise to RGCs. They created a poly(ethylene-co-vinyl acetate) scaffold with parallel grooves using nano-imprinting lithography, considering the rapidity, cost-effectiveness and accuracy of 3D printing technology. After that, the OVs were placed on the scaffold, and RGC axons and neurite growth were observed. The scaffold was able to support the extension of long and organized axons that were functional, and they also showed that the generated scaffold enhanced the growth of RGCs derived from a LHON patient [136].

In 2019, Chen and colleagues employed Tanaka’s protocol [84] to obtain retinal ganglion progenitors from iPSCs. After that, they used an electrospinning technique to fabricate a biodegradable Matrigel-coated polybenzyl glutamate scaffold where the progenitors were cultured. During its degradation, the scaffold released glutamate, which is a known neuron stimulant serving as a chemical guidance cue of neurite alignment and outgrowth. They demonstrated that the neurite density and length were significantly increased in cells grown in the scaffold, which may promote a higher differentiation efficiency towards the RGC lineage [137]. Later that year, Yang’s group employed organic photovoltaic material poly-3-hexylthiophene (P3HT) to create a solar cell-like scaffold, since it is able to generate an electric current upon photoelectric stimulation. They used this device for culturing retinal ganglion progenitors from the differentiated OVs. The RGCs grown on the device exhibited more packed neurites, indicating enhanced differentiation efficiency [138].

The combination of tissue engineering and microfluidics has led to the emergence of microphysiological systems, specifically organ-on-a-chip platforms, which represent another step towards the development of a dynamic 3D in vitro system from iPSCs. This technology integrates microscale human tissues into a microfluidic environment, providing the physiological information of complex interactions between cells. Importantly, it enables a direct and highly precise control of nutrients delivery or mechanical stability [139]. In the context of the retina, Achberger et al. developed a physiologically relevant 3D in vitro model of the human retina by combining human iPSC (hiPSC)-retinal organoids with hiPSC-derived RPE in a retina-on-a-chip [140]. This system integrated more than seven different essential retinal cell types in a highly stable and controlled environment, with the possibility of fully recapitulating the complex architecture and physiology of the human retina. It was composed of two polymer layers: the top layer held both the retinal organoids and the RPE, and the bottom one acted like a channel for vasculature-like perfusion, enabling a constant supply of nutrients and compounds. Both layers were separated by a thin, porous membrane simulating the endothelial barrier [140]. This innovative platform is extremely versatile and has the potential to promote drug development and provide new insights into the underlying pathology of optic neuropathies.

## 4. Perspectives and Concluding Remarks

Undoubtedly, in the last years, significant progress has been made on RGC generation from iPSCs with both 2D and 3D approaches, thanks to the knowledge of in vivo retina development. The feasibility of obtaining a patient’s specific iPSC-derived RGCs holds huge potential in providing new insights of the mechanisms underlying RGC loss and, likewise, to developing novel therapeutic options. On the one side, several models for different mutations causing glaucoma, LHON or optic atrophy have been already devised. Moreover, there is evidence that these models represent an interesting platform for drug screening, since some molecules have been found to be able to rescue the affected phenotype in patients’ iPSC-derived RGCs. This would open up the way for future high-throughput screening studies, with the aim of identifying many compounds in a shortened time, accelerating the drug discovery process.

The ultimate goal would be the translation to the clinics of the differentiated RGCs from iPSCs. These RGCs could be employed for cell replacement therapy in patients with optic neuropathies, and they could also be combined with gene therapy in order to correct the genetic defect before the transplantation. At the moment, there are no examples of ongoing clinical trials using RGCs generated from iPSCs, but perhaps the recent arrival of scaffold technology is helping us become much closer to the first one. As previously mentioned, a Chinese research group obtained very promising results. They were able to achieve the development of a biodegradable PLGA scaffold capable of enhancing RGC differentiation and survival. This scaffold provided the appropriate physical support for the axons to regenerate. They transplanted it into rabbit and monkey retinas as a proof-of-concept, and the graft was partially attached to the retinas, showing to be safe after three months [135].

Within the field of ophthalmological diseases, the first iPSC clinical trial was conducted for age-related macular degeneration (AMD). It was initiated in 2014 when researchers at the Riken Center (Japan) grafted an autologous iPS-RPE cell sheet in a patient’s retina with AMD [141]. The transplant demonstrated its safety, and the progression seemed favorable one year later, although, unfortunately, the trial had to be suspended due to a genetic copy number alteration in the iPSCs of the second patient enrolled [142]. The establishment of a clinical-grade iPSC bank from healthy HLA homozygous donors at the Centre for iPS Cell Research and Application (CiRA) in Japan led to the initiation of another clinical trial in 2017 [143]. HLA-matched allogeneic iPS-RPE cells were grafted to AMD patients, and, although the cell delivery strategy must be further optimized, one year later, the RPE grafts survived and were safe [144].

Regarding RGCs, the development of improved differentiation protocols is required to attain a satisfactory generation efficiency and purity of the RGC population for the final translation to the clinics. That means that the process demands additional optimizations to accomplish a simple and reproducible protocol, with high efficiency and minimal variation in each step. Apart from that, more studies are needed about RGC integration in the ganglion cell layer and their connection with amacrine and bipolar presynaptic neurons, as well as the axon extension towards the optic nerve finally targeting the central nervous system.

Despite the challenges that still have to be addressed, the huge progress achieved in the last years in iPSC-based approaches for the study of hereditary optic neuropathies has created the possibility of finding a treatment for these patients in the near future.

## Figures and Tables

**Table 1:** Retinal ganglion cell differentiation protocols from human induced pluripotent stem cells (iPSCs): 2D approaches.

Authors	Differentiation Approach	Differentiation Factors	Culture Matrix	Markers for RGC Identification	Isolation Method	Time (d)	Efficiency	Ref.
Riazifar et al., 2014	EBs, neural rosettes, neurospheres, RGCs	FBS, DAPT	Gelatin Laminin	Islet-1, TUJ-1, γ-synuclein, BRN3A, THY1	N/A	40	20–30%	[83]
Tanaka et al., 2015	EBs, neurospheres, RGCs	IWR-1e, FBS, Matrigel, SAG, CHIR99021, N2, retinoid acid, BDNF	Poly-D-lysine/laminin	Islet-1, Tuj-1, γ-synuclein, Brn3b, Math5	N/A	35	-	[84]
Ohlemacher et al., 2016	EBs, neural rosettes, neurospheres, RGCs	N2, FBS, B27, BDNF	Poly-L-ornithine/laminin	ISLET1, BRN3, HUC/D, MELANOPSIN, TAU, MAP2, RBPMS	N/A	40	36%	[86]
Teotia et al., 2017	RPCs (Lamba et al., 2010), RGCs	RPCs: Noggin, Dkk-1, IGF-I RGCs: Shh, FGF8, DAPT, follistatin, cyclopamine, BDNF, NT4, CNTF, forskolin, cAMP	Matrigel	Islet-1, Tuj-1, Brn3, Thy1, Atoh7	N/A	36	20–30%	[87]
Lee et al., 2018	EBs, neural rosettes, RGCs	Dorsomorphin, SB431542, XAV939, IGF-I, N2, B27 without vitamin A, bFGF, DAPT	Matrigel Poly-D-lysine/laminin	ISLET1, TUJ1, BRN3B, THY1, MATH5	N/A	40	45%	[88]
Chavali et al., 2020	RPCs, RGCs	RPCs: B27 without vitamin A, N2, XAV939, SB431542, LDN193189, nicotinamide, IGF-I, bFGF, CHIR99021, RGCs: Shh/SAG, FGF8, follistatin, cyclopamine, DAPT, forskolin, cAMP, BDNF, NT4, CNTF	Matrigel (growth factor reduced)	TUJ1, BRN3A, BRN3B, γ-synuclein, THY1, MAP2, RBPMS	MACS (CD90.2 micro beads)	40	95%	[89]

EB, Embryoid Bodies; FBS, Fetal Bovine Serum; SAG, Sonic Hedgehog Agonist; DAPT, N-(N-(3,5-difluorophenacetyl)-L-alanyl)-S-phenylglycine t-butyl ester; RPCs, Retinal Progenitor Cells; BDNF: Brain-Derived Neurotrophic Factor; RGCs, Retinal Ganglion Cells; bFGF, Basic Fibroblast Growth Factor; cAMP, Cyclic Adenosine Monophosphate; CNTF, Ciliary Neurotrophic Factor.

## Data Availability

Data sharing not applicable. No new data were created or analyzed in this study. Data sharing is not applicable to this article.
